# Finite element and deformation analyses predict pattern of bone failure in loaded zebrafish spines

**DOI:** 10.1098/rsif.2019.0430

**Published:** 2019-11-06

**Authors:** Elis Newham, Erika Kague, Jessye A. Aggleton, Christianne Fernee, Kate Robson Brown, Chrissy L. Hammond

**Affiliations:** 1 School of Arts, Woodland Road, Bristol, UK; 2 The School of Physiology, Pharmacology and Neuroscience, Biomedical Sciences, University of Bristol, Bristol BS8 1TD, UK

**Keywords:** finite element, zebrafish, spine, mechanics, deformation, geometric morphometrics

## Abstract

The spine is the central skeletal support structure in vertebrates consisting of repeated units of bone, the vertebrae, separated by intervertebral discs (IVDs) that enable the movement of the spine. Spinal pathologies such as idiopathic back pain, vertebral compression fractures and IVD failure affect millions of people worldwide. Animal models can help us to understand the disease process, and zebrafish are increasingly used as they are highly genetically tractable, their spines are axially loaded like humans, and they show similar pathologies to humans during ageing. However, biomechanical models for the zebrafish are largely lacking. Here, we describe the results of loading intact zebrafish spinal motion segments on a material testing stage within a micro-computed tomography machine. We show that vertebrae and their arches show predictable patterns of deformation prior to their ultimate failure, in a pattern dependent on their position within the segment. We further show using geometric morphometrics which regions of the vertebra deform the most during loading, and that finite-element models of the trunk subjected reflect the real patterns of deformation and strain seen during loading and can therefore be used as a predictive model for biomechanical performance.

## 1. Introduction

The spine consists of a repeated pattern of motion segments (MSs) of bony vertebrae separated by intervertebral discs (IVDs) that enable movement. Back pain and IVD degeneration affect millions of people worldwide [1,2], and vertebral compression fractures are a frequent feature of osteoporosis [3]. Biomechanical pathologies of the spine are underpinned by genetic, physiological and environmental pathways that together damage IVD, muscle and the bone, changing the mechanics of the system.

**Figure 1.**
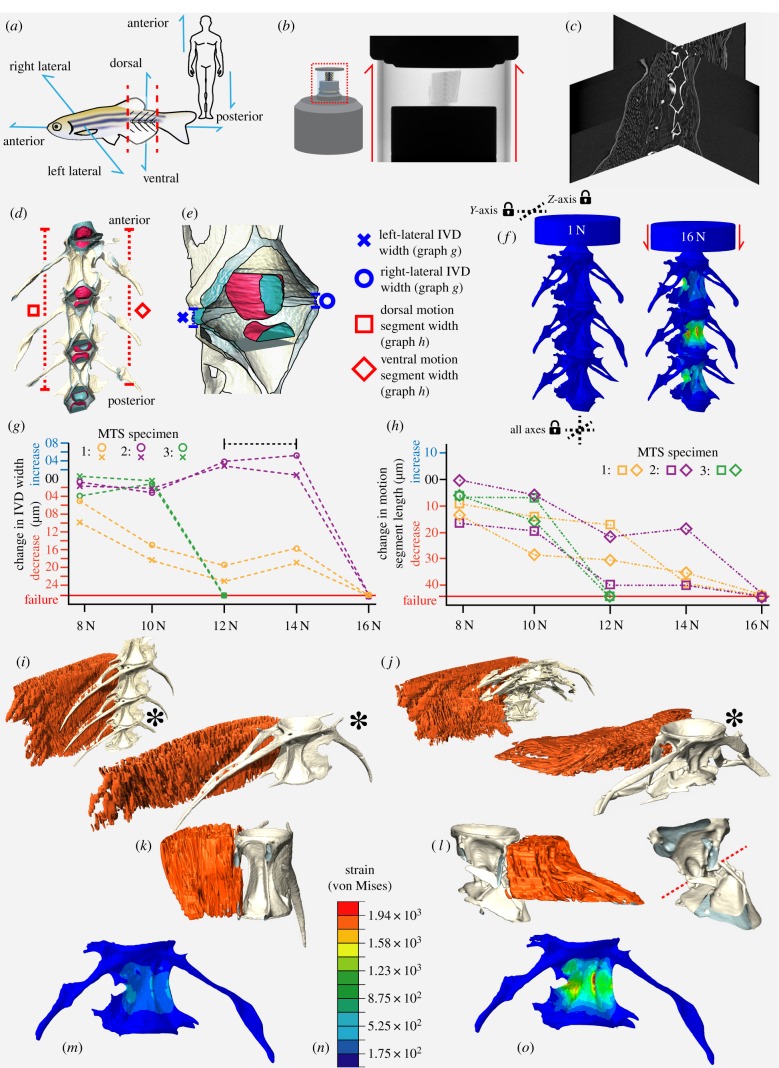
*Ex vivo* spine loading leads to MS failure in a region of high strain predicted by FEA. (*a*) Schematic of zebrafish MS dissection. (*b*) MTS schematic and X-radiograph. (*c*) Orthogonal reconstruction slices showing vertebrae and associated soft tissue. (*d*) Three-dimensional reconstruction of the FEA model with colours reflecting different materials. (*e*) Details of the nucleus-pulposus (pink) and annulus fibrosis (blue) from (*d*) showing linear measurements of IVD thickness. (*f*) Predicted compressive deformation and strain map from FEA; dashed lines indicate axes in which boundary conditions were established. (*g*) Changes to IVD width measurements (bracketed dashed line highlights IVD elastic rebound) and (*h*) changes in MS length with increasing load for the three MTS specimens; symbols correspond to those in (*d*,*e*). Values are the absolute values presented in table 1, relative to their value at 1 N. (*i*,*j*) Reconstructions of MTS specimen 1 compressed to 10 N (*i*) and 16 N (*j*) with central vertebra indicated by asterisk in each. (*k,l*) Antero-posterior cross-sections of the central vertebra at 10 N (*k*) and 16 N (*l*). Muscle segmented in red, and bone in grey in (*i*–*l*). Red dashed line in (*l*) denotes the angle of fracture at the vertebral centrum. (*m*,*o*) FEA strain maps at 10 N (*m*) and 16 N (*o*). Scale shown in (*n*). (Online version in colour.)

Animal models, typically rodents, are frequently used to study mechanisms of spinal pathology [4]. However, quadrupeds are disadvantageous for studying the human spine as gravitational load acts perpendicular to their axial skeleton. Zebrafish are increasingly used as a model for human disease, due to their genetic tractability. Unlike quadrupeds, but similar to humans under gravity (figure 1*a*), their spine is antero-posteriorly loaded as a result of swimming through viscous water [5]. Zebrafish are well established as models for skeletogenesis, pathology and ageing [6], and develop spinal pathologies in response to altered genetics [7] and ageing [8]. However, the biomechanics of the zebrafish spine are comparatively poorly characterized.

**Table 1.** Scanning schedule for specimens analysed using the MTS, and their linear morphometric measurements and PCA scores of landmark deformation data measured using 3D-GM. Zero distance values indicate contact between vertebrae.

				linear IVD proxy measurements (μm)								3D-GM PCA scores					
				IVD between top and middle vertebra				IVD between middle and bottom vertebra				anterior vertebra		central vertebra		posterior vertebra	
specimen	compressive load (N)	left-lateral motion segment length (μm)	right-lateral motion segment length (μm)	left-lateral central distance	right-lateral central distance	ventral central distance	dorsal central distance	left-lateral central distance	right-lateral central distance	ventral central distance	dorsal central distance	PC1 (antero-posterior deformation)	PC2 (lateral deformation)	PC1 (antero-posterior deformation)	PC2 (lateral deformation)	PC1 (antero-posterior deformation)	PC2 (lateral deformation)
MTS 1	1	2116	2128	37.8	39.2	12.15	20.72	74.42	40.8	15.43	8.94	−0.019	0.003	−0.011	0.001	−0.021	0.008
	5	2113	2122	36.97	37.15	10.39	19.17	65.38	36.72	12.37	6.43	−0.015	0.008	−0.006	−0.009	−0.017	0.004
	8	2104	2109	34.17	31.75	7.51	19.89	55.51	31.67	0	4.26	−0.002	−0.003	0.000	0.000	−0.006	−0.002
	10	2099	2094	31.13	31.99	2.95	14.05	46.95	21.43	0	4.43	−0.001	−0.008	0.002	0.012	−0.003	−0.003
	12	2096	2092	29.42	22.42	0	12.43	42.19	17.09	0	0	0.002	−0.013	0.005	0.008	0.007	−0.010
	14	2074	2085	31.47	25.82	0	18.54	46.37	21.05	0	0	0.034	0.005	0.014	−0.004	0.013	0.009
	16	FAILURE	FAILURE	FAILURE	FAILURE	FAILURE	FAILURE	FAILURE	FAILURE	FAILURE	FAILURE	FAILURE	FAILURE	FAILURE	FAILURE	FAILURE	FAILURE
MTS 2	1	2364	2346	24.65	13.86	6.8	10.4	17.25	15.38	3.2	6.85	−0.026	0.002	−0.014	0.002	−0.015	0.001
	5	2356	2344	21.04	12.56	5.72	9.98	16.73	14.12	0	0	−0.002	−0.006	−0.005	−0.002	−0.013	0.001
	8	2339	2344	18.21	12.21	0	0	15.04	13.53	0	0	0.002	−0.008	−0.001	0.011	−0.010	0.002
	10	2336	2338	16.37	9.56	0	0	14.54	10.93	0	0	0.006	−0.008	0.001	0.005	0.005	−0.015
	12	2316	2323	17.2	12.51	9.76	7.82	19.55	17.99	4.5	7.47	0.006	0.008	0.002	0.000	0.012	−0.006
	14	2316	2326	18.94	17.53	11.92	9.4	17.52	19.32	9.5	12.34	0.011	0.004	0.004	−0.004	0.015	0.008
	16	FAILURE	FAILURE	FAILURE	FAILURE	FAILURE	FAILURE	FAILURE	FAILURE	FAILURE	FAILURE	FAILURE	FAILURE	FAILURE	FAILURE	FAILURE	FAILURE
MTS 3	1	1832	1829	15.54	11.67	0	0		15.15	0	0	−0.013	0.002	−0.012	0.007	−0.005	−0.010
	5	1827	1822	14.23	10.28	0	0	15.39	14.2	0	0	0.001	−0.010	−0.001	0.000	0.007	−0.006
	8	1820	1816	13.97	7.75	0	0	15.78	10.26	0	0	0.005	0.010	0.001	−0.012	0.013	0.005
	10	1821	1807	13.31	7.82	0	0	14.97	13.15	0	0	0.007	−0.002	0.013	0.005	0.019	0.008
	12	FAILURE	FAILURE	FAILURE	FAILURE	FAILURE	FAILURE	FAILURE	FAILURE	FAILURE	FAILURE	FAILURE	FAILURE	FAILURE	FAILURE	FAILURE	FAILURE
	14	FAILURE	FAILURE	FAILURE	FAILURE	FAILURE	FAILURE	FAILURE	FAILURE	FAILURE	FAILURE	FAILURE	FAILURE	FAILURE	FAILURE	FAILURE	FAILURE
	16	FAILURE	FAILURE	FAILURE	FAILURE	FAILURE	FAILURE	FAILURE	FAILURE	FAILURE	FAILURE	FAILURE	FAILURE	FAILURE	FAILURE	FAILURE	FAILURE

Finite-element analysis (FEA) has proven a pivotal tool in the study of biomechanical subjects [9], and offers a method for biomechanically characterizing the zebrafish spine, including intact MSs. This technique digitally models an object of known material properties using a series of linked nodes of known number and geometry, that can be subjected to a wide variety of forces outputting the predicted geometry, strain and deformation. Results can be validated by comparison with the results of loading experiments in which a sample is loaded *ex vivo* [10,11]. FEA has been used in zebrafish to test contributions of shape and material properties in joint morphogenesis [12,13] and to study strain patterns in a single vertebra [14].

Here, we describe a novel integrated experimental platform that brings together imaging, modelling and real-world validation to explore the biomechanics of intact zebrafish spinal MSs. We generated an FEA model of the spine, which we validated with a loading experiment using a high-precision material testing stage (MTS) under set loading regimes using micro-computed tomography (µCT). Three-dimensional geometric morphometrics (3D-GM) was used to explore patterns of deformation seen in each vertebra during loading. Comparison of results demonstrated that our FEA model accurately predicted the relative patterns of deformation and strain experienced by real samples loaded *ex vivo*.

## 2. Methods

### 2.1. Zebrafish samples

One-year-old, wild-type (WT) zebrafish were fixed in 4% paraformaldehyde and dehydrated to 70% EtOH. MSs were acquired by making two cuts in the trunk, between the morphologically homogeneous vertebrae 18 and 24 of a total of 33 vertebrae [5] (figure 1*a–c*).

### 2.2. *In vitro* vertebral loading experiment

Loading experiments were conducted using a custom-built material testing stage (MTS2) in a Bruker SKYSCAN 1272 µCT system. Radiographic visualization of each MS (*n* = 3) was performed and if required, vertebrae were trimmed to retain three complete vertebrae and associated IVDs (figure 1*b*–*d*). Samples were stabilized (anterior-up) in the MTS2 using cyanoacrylate glue. The MTS2 was programmed to perform a sequential series of seven scans at a series of increasing loads (table 1), using 60 keV X-ray energy, 50 W current, 5 µm isotropic voxel size and a 0.25 mm aluminium filter. A total of 1501 projections were collected during a 180° rotation, with 400 ms exposure time. Reconstructions were performed using NRecon (v. 1.7.1.0). Surfaces of vertebrae, muscle and IVDs in each dataset were generated using Avizo (Avizo v. 8; Vizualisation Sciences Group) (figure 1*c*–*e* and table 1) and linear measurements of IVDs and MS lengths made using the ‘3D Measurement’ tool. Vertebrae surfaces were further processed in Meshlab (table 2).

**Table 2.** Methodology tree for image processing and analysis.

analysis	step	software	input file	toolkit/function	substage			output file
MTS	1. model creation	Avizo 8.0	reconstruction 16-bit tiff stack of single load step	edit new label field	a. separate segmentation of individual vertebrae using magic wand tool (minimum histogram grey-scale value set at 32 000)			.stl surface file
				generate surface	b. generation of vertebra surface from segmentation using generate surface tool (unconstrained smoothing with a value of two)			
					c. save file as .stl surface file			
	2. model simplification	Meshlab 2016	.stl surface file	quadratic edge collapse deformation	a. simplify .stl by a factor of 10; target number of faces = 10 000; percentage reduction = 0.1; quality threshold = 0.9			.ply file
					b. export simplified mesh as .ply file			
	3. 3D-geometric morphometrics	R; Geomorph package	.ply files	read.ply	a. import .ply file for each compressive load of a single vertebra (from 1 N to last load before failure)			.csv file
				buildtemplate	b. build landmark template of 22 landmarks and 300 surface sliding semi-landmarks along the surface of the 1 N load dataset			
				digitsurface	c. assign the same 22 landmarks for each other dataset			
				gpagen (ProcD = FALSE)	d. perform Procrustes analysis for landmark coordinates			
				plotTangentSpace	e. perform PCA on Procrustes coordinates			
				write.csv	f. save PCA scores to .csv file			
				plotRefToTarget (mag = 10)				
					g. plot vector map of deformation, with vectors magnified by an order of 10			1. PCA scores; 2. vector plot
FEA	1. model creation	Avizo 8.0	1. native state reconstruction 16 bit tiff stack 2. contrast-enhanced reconstruction 16 bit tiff stack	edit new label field	a. find analogous vertebral region to the motion segments analysed in MTS			.stl surface files of individual motionsegment elements
					b. segment bone in the native state reconstruction			
					c. segment bone and IVD in the contrast-enhanced reconstruction			
				three-dimensional (3D) image registration using ‘Algebra’ tool	a. find analogous region of characteristic vertebral morphology in both label fields using the ‘Orthoslice tool’			
					b. manually adjust the volumes in 3D space using the ‘Volume edit’ tool until they overlap precisely in all three dimensions and save their new position in 3D space			
					c. combine both label fields using the ‘Algebra’ tool			
					d. edit new label field to save individual vertebrae and IVDs as separate materials			
				generate surface	e. generation of surface from each individual material using generate surface tool (unconstrained smoothing with a value of two)			
					f. save each material as .stl file			
		ScanIP (with CAD plugin)	.stl surface files of individual motion segment elements	import .stl	a. import individual .stl files			.inp file
				surface to mask	b. convert all surfaces to masks			
				create FE model	c. incorporate all surfaces into FE model			
				model configuration	d. assign material properties to materials			
					**material**	**Young's modulus (GPA)**	**Poisson's ratio**	
					stainless steel^a^	200	0.3	
					vertebral bone^b^	20	0.3	
					anulus fibrosus^c^	4.2	0.25	
					nucleus pulposus^d^	1.72	0.45	
				full FE	generate full FE model and save as an Abaqus input file (.inp)			
	2. compression simulation	Abaqus	.inp file	module: step	a. create seven load steps, all ‘static, general’, to replicate the seven MTS load steps, switching the ‘NIgeom’ option on			.odb Abaqus output database
				module: load	a. create a custom coordinate datum system (CSYS) so that the *x*-axis aligns with the antero-posterior axis of the motion segment			
					b. create boundary conditions in all three axes of CSYS for elements along the posterior edge of the posterior-most IVD			
					c. create boundary conditions in the *y* and *z* axes for elements along the anterior face and the rim of the stainless-steel compressive plate			
					d. create mechanical load of 1 N concentrated force along the *x*-axis for the central node of the anterior face of the compressive plate			
					e. copy this load into each subsequent step, changing the force to that of the corresponding step in the MTS analysis, deactivating each load in the steps they are not required			
				module: job	a. create and submit job			
					b. visualize the maximum deformation			
	3. analysis	Abaqus	.odb Abaqus output database	module: visualization	a. save the image for each loading step			1. images of deformation and strain patterns under increasing loads; 2. stl surface files of deformed motion segment under each compressive load
					b. repeat for von Mises strain			
					c. export deformed meshes of every loading step as .stl surface files			
		Meshlab 2016	.stl surface file	quadratic edge collapse deformation	repeat method used for MTS analysis			.ply file
		R; Geomorph package	.ply files	Geomorph package	repeat method used for MTS analysis			1. PCA scores; 2. vector plot

^a^Material properties from the Engineering Toolbox (www.engineeringtoolbox.com; accessed 18 June 2019).

^b^Material properties from Ofer *et al*. [14].

^c^Material properties assessed using atomic force microscopy of joint cartilage (R. Harniman 2018, personal communication).

^d^Material properties from Panzer & Cronin [15].

### 2.3. Finite-element analysis

An MS surface mesh was created based on a 1-year-old WT specimen µCT scanned using a Nikon XTH 225ST μCT system as described under two conditions: (a) native state and (b) contrast-enhanced following 14 day incubation in 2.5% phosphomolybdemic acid [16]. Scan (a) was used to segment vertebrae (V18–V24), and scan (b) to segment IVDs. The resulting binary labels from scans (a) and (b) were saved as 8-bit tiff stacks, manually registered in 3D space in Avizo (‘Trackball’ tool) and algorithmically combined (‘Algebra’ tool), creating a single volume of separate materials representing three vertebrae and four IVDs (figure 1*d,e* and table 2). A 500 µm thick cylinder was created contacting the anterior-most IVD perpendicular to the model axis, to mimic the stainless-steel compressive plate and distribution of forces applied during loading (figure 1*f*).

The complete vertebral surface mesh was imported into Simpleware ScanIP (v. 2018.12, Synopsys Inc.) to create an FE model. The model consisted of 1 054 187 linear tetrahedral elements joined at 257 392 nodes comprising four material types: vertebral bone, annulus-fibrosus, nucleus-pulposus and stainless steel (figure 1*d*–*f*, table 2). The model was analysed in Abaqus (2018 version). A custom datum coordinate system was created centred on the antero-posterior axis of the model, and a concentrated force applied to the central node of the anterior face of the compressive plate. This loading case was repeated in each of seven steps of a multi-step analysis, with load values matching the increments applied in the MTS (table 1). The model was constrained in two locations using boundary conditions, at the base of the posterior-most IVD (constrained in three axes) and at the top of the compressive plate (constrained in two axes), allowing movement along the model's antero-posterior axis (figure 1*f*). Deformed meshes from each step were exported as surface files and analysed using 3D-GM for quantitative comparison between relative and absolute patterns of deformation predicted by FEA and observed in MTS data.

### 2.4. Three-dimensional geometric morphometrics

**Figure 2.**
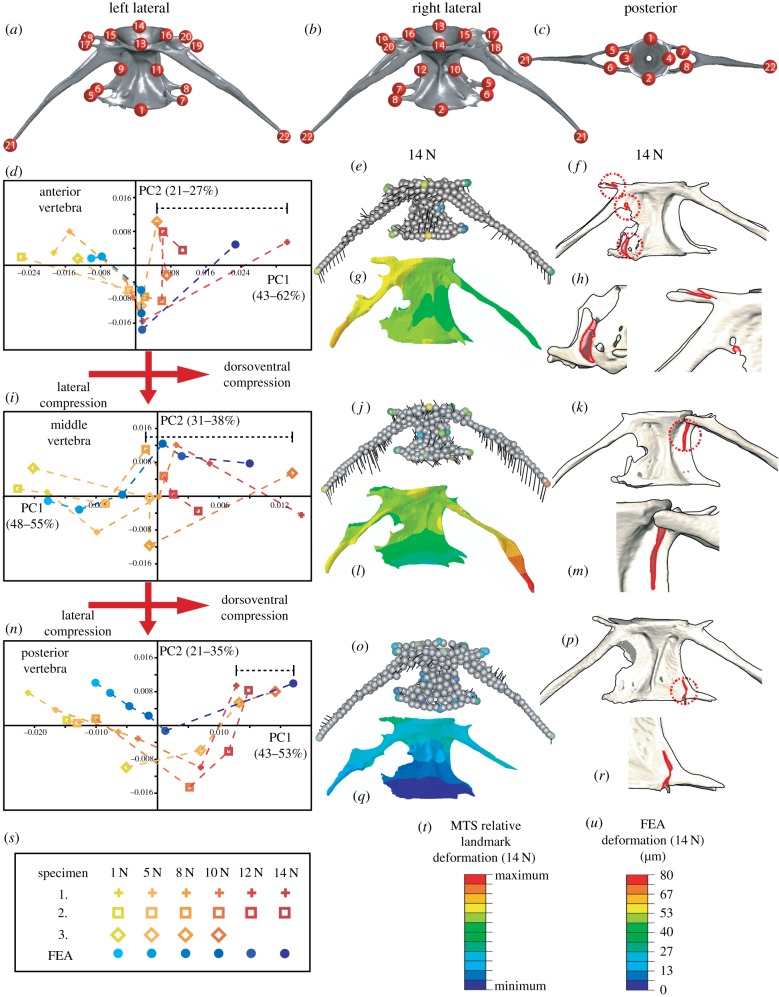
Finite element and geometric morphometric analyses model deformation patterns prior to failure (*a*–*c*). Landmarks assigned for 3D-GM analysis. (*d*,*i,n*) Results of PCA of landmark deformation under increasing compressive loads for each specimen, and deformation predicted by FEA (key in (*s*)). Black bracketed lines indicate reduced lateral compression. (*e*,*j*,*o*) Three-dimensional vector plots with black line vectors representing the direction of landmark deformation and colours highlighting the extent of landmark deformation for each vertebra in specimen 1 (vector scales magnified by 10; colour scale in (*t*)). (*g*,*i*,*q*) Deformation maps predicted by FEA (scales presented in (*u*)). (*f*,*h*,*k*,*m*,*p*,*r*) Examples of fractures (outlined in red for clarity) occurring at compressive loads before failure; corresponding with deformation patterns predicted in FEA and seen *ex vivo.* (Online version in colour.)

Three-dimensional geometric morphometrics analysis of vertebral deformation was performed using the ‘Geomorph’ package for the R statistics software [17]. For each loading experiment, we used the first scan (1 N load) to create a template of 3D coordinates for 22 fixed three-dimensional landmarks (figure 2*a*–*c*) linked by 300 surface sliding semi-landmarks (using the ‘buildtemplate’ function). By assigning the same landmarks in each scan (using the ‘digitsurface’ function), we compared the first scan with subsequent scans of the same vertebra using generalized Procrustes analysis (allowing semi-landmarks to ‘slide’ in order to remove arbitrary spacing). Resulting shape variables were subjected to principal component analysis (PCA) to identify the principal patterns of variation between scans of the same vertebra, and isolate trends in deformation with increasing compressive load.

## 3. Results and discussion

### 3.1. Vertebral motion segments fail under loading of 12–16 N at positions of maximum von Mises strain

To test the range of compressive loads that the MS could resist until failure, we subjected an MS to exponentially increasing compressive forces from 1 to 100 N. This specimen failed at 16 N whereupon the central vertebra fractured mid-centrum. A primary loading regime between 1 and 16 N was thus established (table 1) for the three primary specimens; occupying the elastic, plastic and failure regions of the compressive loading profile of a typical MS. Failure was considered when at least one vertebral centrum fractured across the axis (e.g. figure 1*j*,*l*). All samples failed between 12 and 16 N upon shallow angle fracture in the central vertebra, with the smallest specimen (specimen 3) failing at the lowest force (figure 1*g*,*h*). This is higher than maximum aquatic forces experienced during swim training by Fiaz *et al*. [5], which reached approximately 9.5 N. Minor differences in mounting orientation created differences in linear deformation between right and left sides, but specimens follow similar patterns. Prior to failure, linear measurements show an increase in IVD antero-posterior thickness (table 1, bracketed dashed line in figure 1*g*), suggesting the IVD acts like a coiled spring that may further contribute to the ultimate strain and failure of the segment when released via small-scale bone fracture (figure 1*h*). The surrounding epaxial musculature showed no obvious deformation or damage until the entire MS failed, at which point muscle fibre organization was lost (figure 1*i*–*l*). Comparison between MTS data and FEA results demonstrated strong spatial correlation between maximum predicted strain and ultimate point of failure in the central vertebra (figure 1*m*–*o*).

### 3.2. Morphometric characterization of vertebral compression is predicted by finite-element analysis

We found characteristic patterns of deformation and strain in response to compressive loading of zebrafish vertebrae. Three-dimensional results from MTS data follow distinct trends for each vertebrae between the three specimens (figure 2*d*,*i*,*n*), showing consistent dorsoventral compression, and lateral compression that is reversed at higher loads potentially due to elastic rebound of the IVD and fracturing along the zygopophyses that occurs at these loads (figure 2). This relative pattern is shared between each specimen, although specimen 3 experiences this at lower loads than specimens 1–2, before failing at 12 N. Fractures are observed where the arches and zygopophyses contact the centrum, at loads that precede the failure of the segment (figure 2*f*,*h*,*k*,*m*,*p*,*r*). Comparison with FEA data (blue points in figure 2*d*,*i*,*n*) suggests that the FE model accurately predicts these patterns (figure 2*d*,*i*,*n*), and that patterns of deformation could explain the first signs of damage prior to failure. In both datasets, the anterior vertebra undergoes most deformation, particularly posterior deformation of the arches (figure 2*e*–*h*). The central vertebrae and arches show strong torsion (figure 2*j*–*m*), increasing through the loading regime leading to the failure of the segment (figure 1*l*,*o*). The posterior vertebra shows the least deformation and is most isotropic in pattern (figure 2*o*–*r*), potentially due to protection offered by the anterior IVDs.

Comparison with *ex vivo* loading of vertebral MSs validates the accuracy of our FEA model for predicting patterns of deformation and strain across these structures. This offers a step towards a digital ‘sandbox’ approach to modelling the effects of genetic, physiological and morphological properties on the reaction and resistance of vertebral MSs to loading. Inputting specific properties of vertebral samples into a validated FE model will allow their effects on the biomechanics of the spine to be quantitatively tested *in silico*, allowing the relative contributions of shape and material properties to be explored and empirically tested. This will aid comparison of mechanical performance between different model systems. As an advantage of the zebrafish system is the wealth of mutants modelling human disease genetics [18], comparisons of mechanical performance between genotype and phenotype will be possible. In the longer term, this approach may give insight into biomechanical aspects of spinal pathology, allowing identification of ‘at risk sites’ in the spine. This could provide a basis for more specific or earlier interventions than those commonly employed.

## Data accessibility

Models are available at: http://www.bristol.ac.uk/staff/researchers/data/
